# FTO Gene rs9939609 is Potentially Associated with Diabetes Related Complications in T2DM Patients

**DOI:** 10.2174/0115733998343753250226081545

**Published:** 2025-04-10

**Authors:** Mazhar Hussain, Akbar Waheed, Asim Elahi, Javed Iqbal

**Affiliations:** 1 Department of Pharmacology, Sheikh Zayed Medical College and Hospital, Rahim Yar Khan, Punjab, Pakistan;; 2 Department of Pharmacology, Riphah International University, Islamabad, Pakistan;; 3 Department of Internal Medicine, South Texas Health System McAllen, Texas, 78503, United States;; 4 Department of Medicine, Sheikh Zayed Medical College and Hospital, Rahim Yar Khan, Punjab, Pakistan

**Keywords:** FTO, T2DM, SNP, diabetes complications, lipid profile, blood pressure, HbA1C

## Abstract

**Introduction:**

FTO gene rs9939609, an obesity susceptible gene, has strong with type 2 diabetes mellitus (T2DM). Studies have also established an association between the FTO gene rs9939609 and cardiovascular disease (CVD). This research investigated the association of this genetic variant with microvascular and macrovascular complications related to diabetes.

**Materials and Methods:**

We performed a cross-sectional analysis involving 140 participants with T2DM and 70 healthy control subjects. The DNA samples were analyzed for the FTO gene variant rs9939609 using ARMS-PCR. FTO gene association with diabetes-related microvascular and macrovascular complications was assessed through multivariate logistic regression, with unadjusted odds ratios (OR) and 95% confidence intervals. A *p*-value below 0.05 was considered statistically significant.

**Results and Discussion:**

The genotypic distribution of the FTO gene variant adhered to Hardy-Weinberg equilibrium in the study participants (*p*>0.05). The AA genotype exhibited a robust association with elevated BMI, HbA1C, SBP, DBP, TGs and decreased HDL-C levels relative to the AT and TT genotypes with (*p*=0.002). FTO genotype frequency increased from AA to AT to TT in both macrovascular (CVD) and microvascular complications (retinopathy, nephropathy, and neuropathy). Moreover, risk allele(A) was also significantly contributed to CVD (*p*=0.001), retinopathy (*p*=0.004), nephropathy (*p*=0.001), and neuropathy (*p*=0.002). AA genotype of the FTO gene rs9939609 showed the tendency to increase the risk of CVD (OR, 1.21; 95% CI, 1.07-1.70; *p*=0.04) and retinopathy (OR, 1.18; 95% CI, 1.02-1.87; *p*=0.001) while no significant changes were recorded in diabetic nephropathy (OR,1.56; 95%CI,1.2-2.43; *p*=0.67) and neuropathy (OR, 2.49; 95%, 1.52-4.1; *p*=0.06).

**Conclusion:**

Our data indicate that the FTO gene variant rs9939609 is linked to an elevated risk of both microvascular & macrovascular complications in individuals with T2DM.

## 1 INTRODUCTION

Diabetes mellitus (DM) is a substantial public health issue. In 2017, about 463 million people are living with this chronic metabolic disorder in the world. Current prediction reveals that the burden of DM will estimate to cross 700 million in 2045 [1]. Asia is the epicenter of diabetes because of the strong influence of genetics on its underlying risk factors. South Asians develop diabetes at early age, lower waist circumference, and lower BMI as compared to other ethnicities [2]. In 2021, 30 million people were living with diabetes in Pakistan, and this number will almost double in 2045. Pakistan ranks third globally in the prevalence of diabetes, following India and China. Pakistan has the highest diabetes prevalence rate (30.8%) in the world in 2021. This places a tremendous burden on the public health system, overwhelming its capacity to address the rapidly growing diabetes epidemic and exhausting critical resources [3].

South Asians are more prone to develop diabetes-related micro-macrovascular complications. South Asians also have higher proportions of mortality due to diabetes-related complications than other nations. South Asians also have more severe diabetes related micro and macrovascular complications than Caucasians [4-6]. The risk of CVD is almost double in individuals with diabetes oppose to those without diabetes. Atherosclerotic cardiovascular disease is the major cause of morbidity and mortality in T2DM patients. There is a 40% risk of developing chronic kidney disease (CKD) in T2DM patients. CKD serves as a risk enhancer for CVD inT2DM patients. Microalbuminuria and macro albuminuria are distinct determinants of CVD risk in T2DM [7-9]. In a global study, there was a high prevalence and incidence of diabetes-related microvascular and macrovascular complications over 3 years. The health concern authorities should take early and sustained interventions for early risk factor modification to mitigate the pressure on the healthcare system [10, 11].

The role of genetics in T2DM is already understood and well-established. Numerous genome-wide association studies (GWAS) have discovered various single nucleotide polymorphisms (SNPs) that are highly correlated with T2DM and its complications. Most common of them are PPARG, IGF2BP2, FTO, WFSI, TCF7L2, and SLC30A8 [12, 13]. The FTO gene is positioned on chromosome 16 and consists of nine exons and many single nucleotide polymorphisms (SNPs). Numerous studies have elucidated the strong association between the FTO gene and a variety of conditions, including physical activity, energy intake, metabolic syndrome, obesity, T2DM, NAFLD, CVD, heart failure, hypertension and various cancers [14-26]. The relationship of this genetic variant with T2DM, especially in the context of BMI is still a matter of debate with conflicting findings in the medical research. Some studies have revealed that FTO gene variants exhibit an independent relationship with T2DM, while other research posits that this association with T2DM mediated through BMI. Similarly, some studies have shown a direct association between the FTO gene and CVD, while others suggest that this association is mediated through risk factors of CVD [20, 22, 25, 27-33].

In light of this, current research was undertaken to explore the relationship of the FTO gene variant rs9939609 with diabetes-related microvascular and macrovascular complications.

## 2 MATERIALS & METHODS

This observational study included 140 individuals with T2DM and 70 healthy controls. Participants were selected from the diabetes clinic at Sheikh Zayed Medical College & Hospital Rahim Yar Khan, Punjab, Pakistan. The diagnosis of T2DM was made based on the International Diabetes Federation (IDF) criteria [34]. The healthy group was selected from the patient’s close relatives. Microvascular and macrovascular complications in T2DM patients were assessed by history, physical examination, medical record and laboratory data. Data about physical activity, smoking, and lifestyle activities were collected on a standard questionnaire. The diagnosis of retinopathy was made by fundus examination after dilating the pupil. Neuropathy was determined in accordance with patient history, physical examination, electromyography and nerve conduction studies. Nephropathy was diagnosed by urine albumin test, albumin/creatinine ratio and serum creatinine. The diagnosis of CVD complications in the form of angina and myocardial infarction was made through history, medical records, ECG, Troponin-T, and angiography findings. BMI between 18.5-22.9 kg/m^2^ was categorized as normal weight, 23-24.9 kg/m^2^ as overweight and ≥ 25 kg/m^2^ as obese according to Asian Pacific guidelines [35]. Patients with a history of pregnancy, lactation, cancer, hepatic dysfunctions, and CNS disorders were excluded from the study. Additionally, any recognized forms of diabetes, including T1DM, gestational diabetes, and secondary diabetes, were explicitly omitted from the study.

The data on the antidiabetic medication duration were also recorded on a structured proforma. The participant’s data were collected on a predesigned proforma after obtaining both verbal and written consent. The perspectives of the research were explicitly communicated to all study subjects. The study duration was six months (from July to December 2022) and was approved by the institutional review board (IRB) under approval number 58/IRB/SCMC/SZH-2020. The study protocols were followed in strict compliance with the standards set forth in the Helsinki Declaration.

Anthropometric parameters, including body weight, height, waist circumference, and BMI, were taken for all study participants. Body weight and height were recorded while standing, wearing light garments, and barefoot. Body weight was assessed using an electronic scale, and height was recorded with a microtoise. Blood pressure was recorded twice, with a 10-minute interval between measurements. The blood samples were collected to analyze biochemical parameters, such as fasting blood glucose (FBG), HbA1c, total cholesterol (TC), serum triglycerides (TGs), LDL-cholesterol, and HDL-cholesterol, following standard guidelines. The FBG was quantified using the conventional glucose oxidase-peroxidase technique with an automated analyzer. The lipid profile was assessed using a biochemical analyzer with an enzymatic method (Hitachi, Tokyo, Japan).

### 2.1 PCR Amplification and Genotyping

A 5 ml of blood was transferred to an EDTA tube for genotyping. Genomic DNA was isolated from white blood cells (WBCs) employing the Promega Wizard^®^ DNA isolation kit, following standard protocols. The DNA was purified by electrophoresis on a 0.7% agarose gel. DNA fragment amplification was carried out by designing specific primers using primer 3 software (Table **1**).

The specific DNA sequence (short oligonucleotide primers) was used to identify the specific FTO gene rs9939609. Genotyping of the FTO gene variant rs9939609 was performed *via* the tetra-primer amplification refractory mutation system polymerase chain reaction (ARMS-PCR) technique. The PCR reaction was performed.

### 2.2 Thermal Cycling Protocols

The double-stranded DNA was first denatured at 95°C for 1-3 minutes, followed by 35 amplification cycles. The final denaturation was performed at 95°C for 1 minute. The annealing step was performed at 65°C, while the extension was performed at 72°C for 1 minute and 30 seconds. A final extension was done at 72°C for 3 minutes. The PCR-amplified products were loaded onto a 1.2% agarose gel stained with ethidium bromide, alongside 5 µl of 25-2000 bp DNA ladder (BIONEER, Korea). The jell was run at 120 volts for 15 minutes, and the amplified product was visualized under UV light. DNA sequence amplification was performed using the ARMS-PCR method, following standard protocols. The amplified products were further confirmed by sequencing.

### 2.3 Statistical Analysis

Data analysis was performed using the Statistical Package for the Social Sciences (SPSS 24). Quantitative data are expressed as mean ± SD, while categorical data are presented as percentages. Allele/genotype frequencies were calculated using the Chi-squared test. The impact of the FTO gene rs9939609 on study parameters was evaluated through ANOVA. The correlation between the FTO gene variant and diabetes-related microvascular and macrovascular complications was analyzed through multivariate logistic regression, with unadjusted odds ratios (OR) and a 95% confidence interval. A *p*-value below 0.05 was interpreted as statistically significant.

## 3 RESULTS

This research included 140 T2DM patients and 70 healthy controls. The demographic traits of the study subjects are illustrated in Table **2**. Most baseline characteristics, including BMI, WC, SBP, DBP, FBS, HbA1C, TC, TG, LDL-C, and HDL-C differed significantly between the two study groups.

Table **3** displays the distribution of genotype and allele frequencies. The genotypic distribution of the FTO gene variant was consistent with Hardy-Weinberg equilibrium in the study population (*p*>0.05). No notable difference in genotype distributions was observed between the study cohorts (*p*=0.22). However, allele frequencies (A *vs.* T) showed a significant statistical difference between T2DM and the healthy group (*p*=0.02).

When the TT genotype of the FTO gene rs9939609 was used as the reference genotype, both the AA and TA genotypes were strongly linked to an elevated risk of T2DM (OR, 1.57; 95% CI, 1.37-3.82) with *p*=0.04 for AA and (OR, 1.87; 95% CI, 1.54-3.56) with *p*=0.03 for TA. Similarly, when the T allele of the FTO gene rs9939609 was used as the reference allele, the A allele demonstrated considerable association with an increased risk of T2DM (OR, 2.86; 95% CI, 1.80-3.70) *p*=0.02 (Table **3**).

We also examined the association between the FTO variant rs9939609 and clinical parameters using a recessive model (AA/(TA+TT). Table **4** presents the relationship of this genetic variant to various study attributes. BMI was categorized according to the Asian Pacific guidelines [35], with thresholds defined as normal weight (18.5-22.9 kg/m^2^), overweight (23.0-24.9 kg/m^2^), and obese (≥25 kg/m^2^)]. In the current research, the AA genotype exhibited a notable correlation with increased obesity, particularly in comparison to the AT and TT genotypes, with a value of (0.001). Similarly, glycemic control was classified according to HbA1C level as good (< 7%), inadequate (7-8%) and poor (>8%) based on the American Medical Association guidelines [36]. Our analysis revealed that the AA genotype was strongly correlated with poor glycemic control in contrast to the AT and TT genotypes (*p*=0.000). Primary hypertension was classified as having a systolic blood pressure (SBP) of 140 mm Hg or higher, or a diastolic blood pressure (DBP) of 90 mm Hg) or higher. The AA genotype exhibited a significant association with both SBP and DBP, surpassing the AT and TT genotypes, with *p*-values of 0.000 for both measurements.

Regarding the lipid profile, the AA genotype was notably associated with elevated triglyceride levels and decreased HDL-C concentrations when compared to the AT and TT genotype, with *p*-values of 0.002 and *p*=0.027, respectively. However, AA *versus* AT/TT genotypes recorded no significant changes in total and LDL-C cholesterol levels.

Table **5** shows the link of the FTO gene rs9939609 with the risk of both microvascular and macrovascular outcomes in patients with T2DM. The FTO genotype frequency increased in order from AA to AT to TT in both macrovascular (CVD) and microvascular complications (retinopathy, nephropathy, and neuropathy) (Fig. **1**). The risk allele (A) was significantly associated with CVD (0.001), retinopathy (0.004), nephropathy (0.001), and neuropathy (0.002). AA genotype of the FTO gene rs9939609 demonstrated a propensity to elevate the risk of CVD (OR, 1.21; 95% CI, 1.07-1.70; *p*=0.04) plus retinopathy (OR, 1.18; 95%CI, 1.02-1.87; *p*=0.001). However, no significant associations were found for diabetic neuropathy (OR, 2.49; 95%, 1.52-4.1; *p*=0.06). and nephropathy (OR, 1.56; 95% CI,1.2-2.43; *p*=0.67).

## 4 DISCUSSION

This study demonstrates a robust link of the FTO gene rs9939609 to both T2DM and its micro-macrovascular complications. While many studies have investigated the association between the FTO gene and T2DM, few have examined its association with diabetes-related microvascular and macrovascular complications. To the best of our knowledge, this is pioneering research in Pakistan investigating the observed associations.

Studies have demonstrated a strong association between the FTO gene rs9939609 and CVD. Several analyses have indicated a direct correlation between the FTO gene and CVD, while others have suggested that the association is mediated through CVD risk factors [20, 25, 30-33]. In our study, patients with T2DM exhibited markedly elevated body mass index(BMI), blood pressure, HbA1C, and TG levels, alongside decreased HDL-C. Additionally, the AA genotype of the FTO gene rs9939609 showed a robust relationship with diabetes-related macrovascular complication (CVD) and retinopathy. The Go-DARTS study [37] showed a strong correlation between the AA genotype of FTO gene rs9939609 and CVD, based on BMI, lipid profile and insulin resistance. Similarly, the Finnish Diabetes Prevention Study (DPS) [38] and a study conducted in Pakistan [39] showed that the AA genotype exhibited a marked correlation with poor glycemic control, effectively doubling the risk of CVD in patients with T2DM.

Increased triglycerides (TGs) and reduced HDL-C levels are closely linked to a higher risk of CVD. The TG/HDL-C ratio is considered a key predictor of both cardiovascular disease and metabolic syndrome [40, 41]. The association between lower HDL-C levels and CVD has also been demonstrated in studies by Mehrdad *et al, *2020 [42], Jalili *et al, *2021 [43], Khella, *et al. *2019 [44], Doney *et al, *2007 [37] and Franczak *et al, *2018 [45]. These studies indicated that individuals possessing the AA genotype (rs9939609) had substantially lower HDL-c levels relative to those carrying the TT genotype. In contrast, no substantial associations were observed with other lipid traits. This supports the evidence that the AA genotype predisposes to CVD by disturbing serum HDL-C levels. Similarly, other research has demonstrated how the risk allele of FTO gene rs9939609 predisposes individuals to CVD by altering the lipid profile [46, 47, 33, 48]. One proposed mechanism is FTO-mediated m6^A^ demethylation, by which FTO genes disrupt the lipid profile. FTO overexpression enhances lipogenesis, accelerates fat accumulation in adipose tissue, promotes lipids synthesis in the liver, and suppresses lipolysis in adipocytes. Upregulation of FTO decreases insulin secretion through an inflammatory pathway, which may predispose individuals to diabetic dyslipidemia [49].

On the other hand, the OPERA study (Oulu Project Elucidating Risk of Atherosclerosis) highlighted a direct correlation between the AA genotype and the incidence of CVD, which was not mediated through any conventional risk factor of CVD [50]. In another study, the FTO gene rs17817449 demonstrated a direct correlation with CVD in Caucasian males, irrespective of age, BMI, and glycemic status [51]. A study conducted in Sweden also yielded similar results [52]. Comparable results were found in a meta-analysis of ten studies conducted on the European population [53].

We also investigated the association between the FTO gene rs9939609 and diabetes-related microvascular complications. Although the frequency of FTO genotype increased in order from AA to AT to TT in diabetic retinopathy, nephropathy, and neuropathy. We observed a notable correlation solely with diabetic retinopathy, while no significant risk was identified in diabetic neuropathy or nephropathy.

A study was carried out within the Czech-Slavonic cohort to investigate the relationship linking the FTO gene variant rs17817449 to the onset of diabetes and its associated complications. This study revealed that the GG genotype frequency was notably more prevalent in T2DM (25.4%) as compared to the control group (16.7%). Moreover, the GG genotype demonstrated a correlation with an increased risk of diabetic nephropathy and neuropathy in T2DM patients. However, no association was detected with diabetic retinopathy upon accounting for chronological age, gender, and BMI [54].

A study was conducted to assess the activity of the FTO gene and its protein concentration in individuals with T2DM, concerning glucose regulation and diabetes-related issues. The study found that the transcriptional activity of the FTO gene and its protein concentrations were strongly associated with the onset and severity of diabetic complications. This increased expression was considered a risk factor for the severity of T2DM [55]. Asimilar study concluded that FTO mRNA and protein concentrations were markedly elevated in T2DM patients relative to healthy controls. This increased expression was positively correlated with diabetes severity. So, it can be used as a diagnostic tool to assess diabetes-related complications [56]. A genome-wide association study (GWAS) involving 10, 912 subjects in Japan showed that the FTO gene rs56094641 showed a significant association with diabetic nephropathy. This significant relationship was maintained after accounting for age, sex, and BMI [57].

A cross-sectional study comprising 236 T2DM patients indicated that the C allele of FTO gene rs7204609 is strongly linked with chronic kidney disease(CKD). However, this association was mediated through waist circumference, BMI, blood pressure, and albuminuria [58]. A study conducted on 551 T2DM patients by Hsiao *et al, *2021 in Taiwan postulated that FTO gene rs8050136 showed a strong association with diabetic retinopathy in all three models (additive, dominant and recessive. After adjustment of study variables *i.e.* waist circumference, HbA1c, SBP, lipid profile, albuminuria, and diabetes duration. FTO gene rs8050136 showed an independent association with diabetic retinopathy risk [59].

40 SNPs in 21 candidate genes were studied to determine micro and macrovascular complications in 503 T2DM patients and 580 control subjects. It was found that FTO, UCP, and CAT genes were significantly associated with diabetic nephropathy and retinopathy [60]. Similarly, 21 SNPs were analyzed in 1784 T2DM patients and 2481 healthy subjects to predict diabetes-associated complications. It was concluded that SNPs of FTO, TCF7L2, and IGF2BP2 were strongly associated with diabetes-related complications (neuropathy, nephropathy, and leg ischemia) [61].

## 5 LIMITATIONS AND FUTURE DIRECTION

The main limitations of our study are its cross-sectional design and small sample size. Further studies with large sample sizes should be conducted to provide broader perspectives. Secondly, longitudinal interventional studies should be undertaken to elucidate the relationship between the FTO gene variant and diabetes-related microvascular and macrovascular sequelae. Moreover, we found no significant effects of anti-diabetic drugs on biochemical parameters with the FTO gene rs9939609 variant in T2DM patients. It would be beneficial to conduct interventional follow-up studies to analyze the effects of antidiabetic drugs on the FTO gene rs9939609 variant in patients with diabetes, as well as in those with both diabetes and CVD. BMI is one of the most important determinants of micro and macrovascular complications in T2DM patients. In our study, 100% of T2DM with the AA genotype were obese and showed a strong association with CVD and retinopathy. That’s why the BMI cut-off value ≥23 kg/m^2^ is suggested for Asians and traditional BMI cut-off value ≥25 kg/m^2^ for the Western cohorts does not apply to the South Asian population. Maintaining a normal BMI according to Asian Pacific Guidelines may reduce CVD risk factors and diabetes-related complications in individuals with type 2 diabetes.

The findings of this research additionally propose that the FTO gene variant rs9939609 could serve as a potential biomarker for detecting and predicting diabetes-related complications in individuals with T2DM, thereby alleviating the burden of T2DM and its related complications.

## CONCLUSION

FTO gene rs9939609 is linked to an elevated risk of diabetes-related micro & macrovascular complications in individuals with T2DM. Recognizing individuals vulnerable to diabetes-related complications is of paramount importance, as the FTO gene could be used as a predictive model to identify T2DM patients at higher risk of developing diabetes-related complications in clinical practice.

## Figures and Tables

**Fig. (1):**
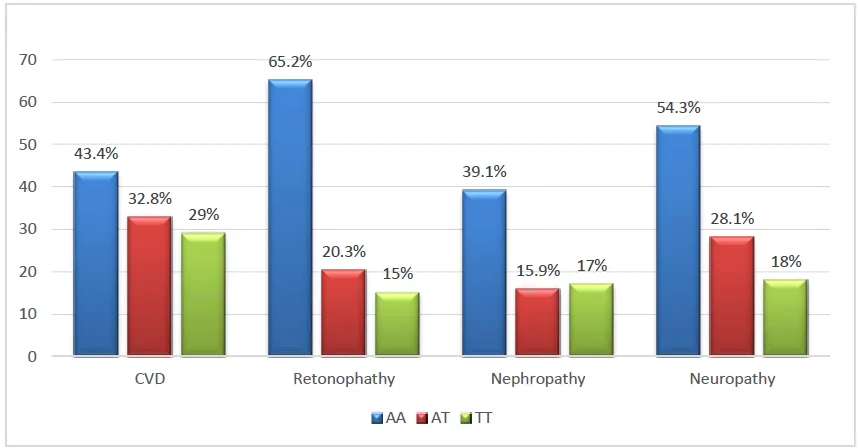
Frequency of diabetic complications with FTO genotype rs9939609.

**Table 1:** The sequences of forward and reverse primers were as follows.

**Primer Name**	**Primer Sequence (5’-3’)**	**Length (bps)**	**Product Length (bps)**
Forward-rs-9939609	5’-TGGTGGTACGCTGCTATGGTTCTA-3’	24	455
Reverse-rs-9939609	5’-CAGCCTCTCTACCATCTTATGTCC-3’	24	

**Table 2:** General characteristics of study groups.

**Parameters**	**T2DM**	**Control**	***p*-value**
Age (years)	48±8.2	43.39±12.462	0.008
Sex (M)	46(65.70%)	45(64.30%)	0.21
History of smoking	51	20	0.25
Family history diabetes	90	37	0.11
BW (kg)	77±11.3	77.91±13.29	0.22
BMI (kg/m^2^)	25.5±1.4	21.10±2.77	0.000
WC (cm)	88.2±8.5	73.73±7.22	0.000
SBP (mm Hg)	122±18.3	113.00±5.98	0.000
DBP (mm Hg)	83.1±9.1	79.93±5.21	0.000
FBG (mg/dl)	120.5±29.3	85.20±13.66	0.000
HbA1c (%)	7.3±0.96	5.32±0.64	0.000
TC (mg/dl)	168.4±23.8	155.96±30.03	0.000
LDL (mg/dl)	132.1±17.1	108.96±20.99	0.000
HDL (mg/dl)	37.1±4.6	42.69±2.36	0.000
TG (mg/dl)	183.4±56.2	101.37±17.43	0.000

**Note: **Data are presented as means ± standard deviation, except gender. *p*-values were calculated using a one-way analysis of variance, except for gender, which was analyzed using Chi-square test.

**Table 3:** Genotype/Allele frequency of FTO gene variant (rs9939609) in various study groups.

**Genotype/Allele**	**T2DM** **140**	**Control** **70**	***p*-value**	OR (95%CI) *
AA	36 (25.71%)	10 (14.30%)	0.22	1.57 (1.37-3.82) *0.04
TA	44 (31.42%)	20 (28.60%)		1.87 (1.54-3.56) *0.03
TT	60 (42.85%)	40 (57.10%)		
A -Allele	58 (82.85%)	20 (28.57%)	0.02	2.86 (1.80-3.70) *0.02
T -Allele	82 (58.57%)	50 (71.42%)		

**Note:** *p* values were calculated using the Chi-square test*p value. AA *vs.* TA & TT genotype, A *vs.* T allele. T2DM, type 2 diabetes mellitus; OR denotes odds ratio; CI represents confidence interval.

**Table 4:** Association of FTO genotypes to study variables in T2DM patients.

**Parameters**	**TT (n=100)**	**TA (n=64)**	**AA (n=46)**	***p*-value**
**BMI**				
**Normal**	30 (30%)	14 (22.0%)	9 (19.56%)	0.000
**Overweight**	60 (60%)	22 (34.37%)	3 (6.52%)	
**Obese**	10 (10%)	28 (44.0)	34 (74.0%)	
**HbA1C**				
**Good (<7)**	51 (51%)	22 (34.37%)	10 (21.73%)	0.000
**Inadequate (7-8)**	39 (39%)	28 (43.75)	3 (6.52%)	
**Poor (>8)**	10 (10%)	14 (21.87)	33 (71.7%)	
**Systolic blood pressure**				
**>140 mmHg**	2 (2%)	4 (6.25%)	28 (60.87)	0.000
**<140 mmHg**	98 (98%)	60 (93.75)	18 (39.1%)	
**Diastolic blood pressure**				
**>90 mmHg**	4 (4%)	4 (6.25%)	33 (71.73)	0.000
**<90 mmHg**	96 (96%)	60 (93.75)	13 (28.26%)	
**Total cholesterol**				
**<200 mg/dl**	65 (65%)	49 (76.56%)	30 (65.21%)	0.22
**>200 mg/dl**	35 (35%)	15 (23.43%)	16 (34.78%)	
**Triglycerides**				
**<150 mg/dl**	72 (72%)	27 (42.18%)	11 (23.91%)	0.000
**>150 mg/dl**	28 (28%)	37 (57.81%)	35 (76.08%)	
**LDL-C**				
**<130 mg/dl**	55 (55%)	30 (46.85%)	21 (45.65%)	0.45
**>130 mg/dl**	45 (45%)	34 (53.12%)	25 (54.34%)	
**HDL-C**				
**<40 mg/dl**	16 (16%)	24 (37.5%)	30 (65.21%)	0.000
**>40 mg/dl**	84 (84%)	40 (62.5%)	16 (34.78%)	

**Note:** *p* values analysis by Chi-square. The comparison of the AA genotype with combined AT + TT genotypes was carried out using a recessive model.

**Table 5:** The correlation between FTO genotypes and the risk of diabetic complications in patients with T2DM.

**T2DM Complications**	**FTO Genotypes**			***p*-value**		**OR(95%CI)^b^**
	**AA (n)**	**AT (n) 64**	**TT (n) 100**	**p^a^**	**p^b^**	
CVD	20 (43.47%)	21 (32.81)	29 (29%)	0.001	0.005	1.21 (1.07-1.70)
Retinopathy	30 (65.21%)	13 (20.3%)	15 (15%)	0.004	0.001	1.18 (1.02-1.87)
Nephropathy	20 (39.1%)	10 (15.93%)	17 (17%)	0.001	0.67	1.56 (1.2-2.43)
Neuropathy	26 (54.34%)	18 (28.12)	18 (18%)	0.002	0.06	2.49 (1.52-4.1)

**Note: **Chi-square test was employed to analyze allele frequency(p^a^) and genotype distribution (p^b^).

## Data Availability

All relevant data have been provided in the main manuscript. The current analyzed data are available from the corresponding author and can be given upon reasonable request by email.

## LIST OF ABBREVIATIONS

T2DM: Type 2 Diabetes Mellitus
CVD: Cardiovascular Disease
IRB: Institutional Review Board
WBCs: White Blood Cells
OR: Odds Ratios
IDF: International Diabetes Federation
DM: Diabetes Mellitus
CKD: Chronic Kidney Disease
SNPs: Single Nucleotide Polymorphisms

## STANDARDS OF REPORTING

STROBE guidelines were followed.
